# Pairwise visual comparison of small RNA secondary structures with base pair probabilities

**DOI:** 10.1186/s12859-019-2902-6

**Published:** 2019-05-29

**Authors:** Serge Léger, Maria Beatriz Walter Costa, Dan Tulpan

**Affiliations:** 1 Department of Animal Biosciences, Centre for Genetic Improvement of Livestock, University of Guelph, Guelph, Ontario Canada; 2 Digital Technologies Research Center, National Research Council Canada, 100 des Aboiteaux St, Moncton, NB E1A7R1 Canada; 3 School of Computer Science, University of Guelph, Guelph, Ontario Canada; 4 Department of Computer Science, TFome Research Group, Bioinformatics Group, Interdisciplinary Center of Bioinformatics, University of Leipzig, Härtelstrasse 16-18, D-04107 Leipzig, Germany

## Abstract

**Background:**

Predicted RNA secondary structures are typically visualized using dot-plots for base pair binding probabilities and planar graphs for unique structures, such as the minimum free energy structure. These are however difficult to analyze simultaneously.

**Results:**

This work introduces a compact unified view of the most stable conformation of an RNA secondary structure and its base pair probabilities, which is called the **C**ircular **S**econdary **S**tructure **B**ase **P**airs **P**robabilities **Plot** (**CS**^**2**^**BP**^**2**^**-Plot**). Along with our design we provide access to a web server implementation of our solution that facilitates pairwise comparison of short RNA (and DNA) sequences up to 200 base pairs. The web server first calculates the minimum free energy secondary structure and the base pair probabilities for up to 10 RNA or DNA sequences using RNAfold and then provides a two panel comparative view that includes CS^2^BP^2^-Plots along with the traditional graph, planar and circular diagrams obtained with VARNA. The CS^2^BP^2^-Plots include highlighting of the nucleotide differences between two selected sequences using ClustalW local alignments. We also provide descriptive statistics, dot-bracket secondary structure representations and ClustalW local alignments for compared sequences.

**Conclusions:**

Using circular diagrams and colour and weight-coded arcs, we demonstrate how a single image can replace the state-of-the-art dual representations (dot-plots and minimum free energy structures) for base-pair probabilities of RNA secondary structures while allowing efficient exploration and comparison of different RNA conformations via a web server front end. With that, we provide the community, especially the biologically oriented, with an intuitive tool for ncRNA visualization.

*Web-server:* *https://nrcmonsrv01.nrc.ca/cs2bp2plot*

## Background

Visual analysis of biological sequences is a crucial step in bioinformatics and computational biology and contributes substantially to biological data interpretation and algorithm development. Traditional representations of RNA secondary structures, for example (such as linear arc diagrams, circular diagrams and planar graphs), present a single state view of a structural conformation of a nucleic acid, which provide fundamental insights into the cellular function of both coding and non-coding RNAs [1]. In reality, an RNA molecule transitions among a variety of energy states due to thermodynamic variations in its environment [2]. Therefore, more advanced and accurate visualization approaches are needed to characterize the whole ensemble of secondary structure conformations for an RNA sequence.

Visualization approaches [3–6] based on predicted RNA secondary structures [7] have been developed to separately address these needs. Single state RNA conformations can be depicted with *planar graphs*, *linear arc diagrams* and *circular diagrams*, based on different ways of representing the RNA backbone.

**Fig. 1:**
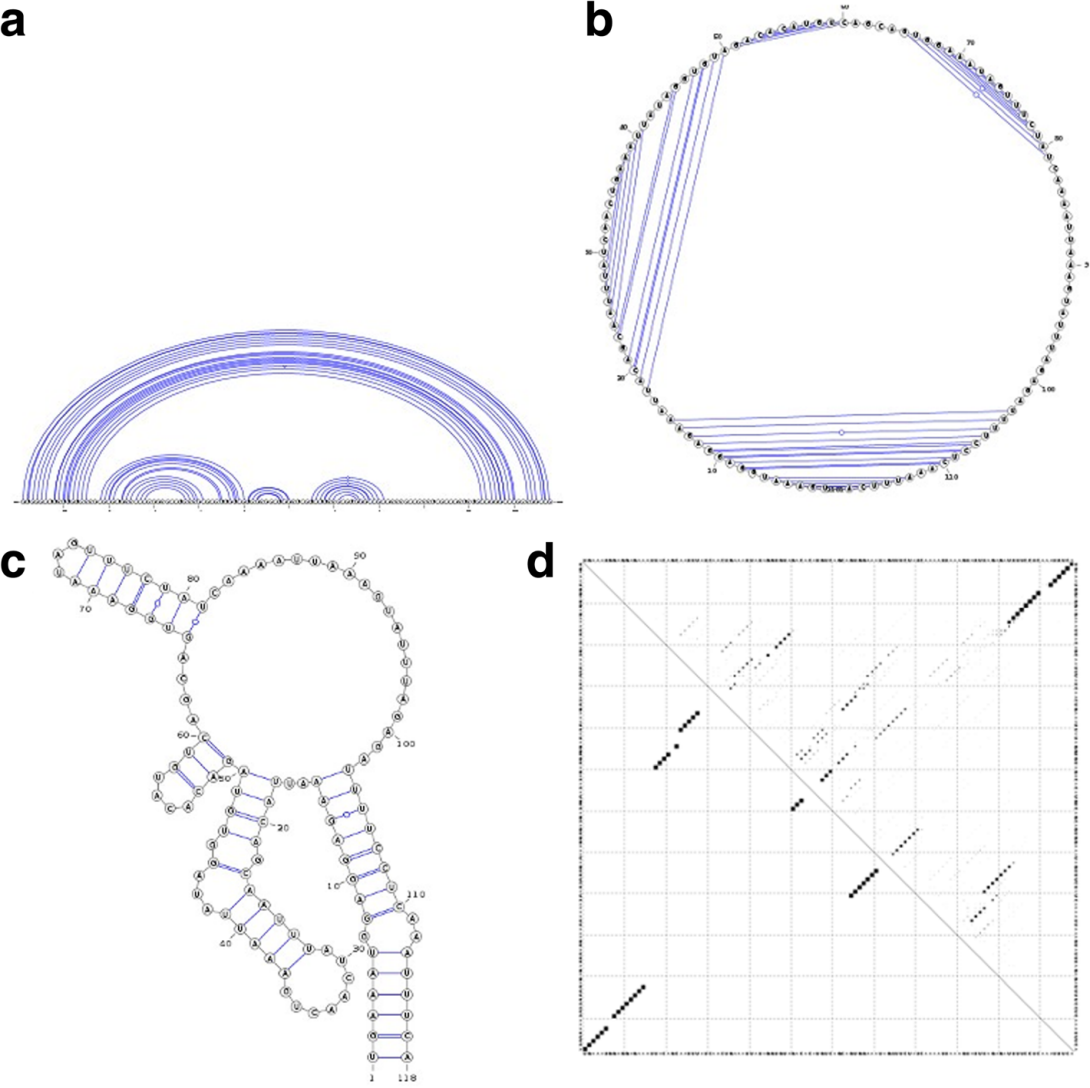
Different representations of an RNA secondary structure. Sub-figure (a) depicts a linear arc diagram, sub-figure (b) a circular diagram, sub-figure (c) a planar graph, and sub-figure (d) a dot-plot diagram for the ancestral ncRNA region of HAR1

In linear arc diagrams (Fig. 1a), the backbone is represented by a straight line. The bases are consecutively placed along the line and paired bases are connected with arcs. To save space and accommodate longer RNA sequences, the backbone can be displayed in a circular format as originally proposed by Nussinov et al. in 1978 [8]. A circular diagram (Fig. 1b) will therefore consist of a circular backbone with bases lined up consecutively along the perimeter of the circle, while chords will connect the paired bases. A planar graph (Fig. 1c) is by far the most used visualization method to date, due to its flexibility and simplicity. It represents the backbone as a convoluted planar curve with predefined distances between neighbouring paired bases and a minimum number of overlaps.

Base-pair probabilities are typically represented with *dot-plots* (Fig. 1d) – bi-dimensional graph representations with positions and bases of the sequence being present on both x and y axis and dots being placed at positions where base pairs occur. In this case, the dot sizes are proportional to the base pair probabilities. An extensive presentation of RNA dot-plots and their usage is explored by Churkin and Barash [9]. Other RNA secondary structure alignment and pairwise comparison methods have been published in the past, most notably RNAforester [10], R-chie [5] and BEAGLE [11]. RNAforester calculates RNA secondary structures alignments using dot-parentheses representations as input and applies a tree alignment model on all sequence secondary structures in a progressive fashion. R-chie focuses on highlighting structure and primary sequence conservation and variation in multiple RNA secondary structures. BEAGLE exploits a new encoding for RNA secondary structure and a substitution matrix of RNA structural elements to perform RNA structural alignments and easily identify structural similarities between RNAs.

In 2013, Aalberts and Jannen [4] introduced the RNAbows diagrams, the first successful attempt to combine two representations of RNA secondary structures with base pair probabilities. It captures both the minimum free energy (MFE) structure and the base pair probabilities into a single image. While their method is versatile and allows for comparison of pairs of RNA secondary structures, their choice of linear arc diagrams places serious constraints on the maximum length of RNA sequences that can be represented (less than 100 bases in a legible format). Moreover, pairwise (bottom-up) comparisons require sequences of equal length that can be horizontally aligned.

Other bioinformatics tools have been recently proposed for visualization or comparison of RNA secondary structures, such as RNA-TVCurve [12] for secondary structure comparisons and TRAVeLer [13] for visualization of structures in the presence of a template. While interesting, they solve different problems and require additional constraints that limit their applicability for both comparison and visualization of RNA secondary structures with base-pair probabilities, such as the need of external structure templates, as is the case of TRAVeLer.

To alleviate these limitations and to propose an alternative tool that is informative and easy to use for biologists, we present the Circular Secondary Structure Base Pairs Probabilities Plot (**CS**^**2**^**BP**^**2**^**-Plot**) – an intuitive visual representation of an RNA secondary structure that includes all possible base pair probabilities. The CS^2^BP^2^-Plot uses a chord diagram layout comprised of two concentric graphical layers representing (i) the RNA sequence and corresponding positions for each base pair (outer layer), and, (ii) the base pair probabilities and MFE base pairings (inner layer).

## Results and discussion

We consider five use cases in this study to further show how the CS^2^BP^2^-Plot can be successfully used as a comparative tool to discover important characteristics of RNA molecules and shed light on pertinent biological questions. The applications we show are very diverse and exemplify how our new tool can be used in studies of evolution, function and engineering of ncRNAs.

### Case study 1: visualization of evolutionary changes in non-coding RNAs

Human accelerated regions (HARs) are sequences in the DNA that have an accumulation of human specific changes [14], which were caused by accelerated rates of nucleotide substitutions in humans, when compared to other vertebrates. Located at the end of chromosome 20 in humans, the HAR1 in particular, shows the highest substitution rate among HARs. The HAR1 is 118 bp long and has 18 substitutions that are specific to the human species. The HAR1 is part of two overlapping long ncRNAs, HAR1F and HAR1R, both of which are expressed very specifically in the brain in a developmental time point that is fundamental for brain formation [14]. Different studies of experimental biology have shown that the 118 bp HAR1 [14–18] folds into a stable secondary structure, which differs between the human species and the other vertebrates. If we consider the structure of non-human species as the ancestral structure, that means that the HAR1 has been kept conserved in non-human species since the last common ancestor and changed only in the human lineage. This is a likely indicator of adaptive evolution acting on the human version of HAR1. To confirm this hypothesis, it is of great importance to compare structures. In this way, we can better understand how the HAR1 evolved in humans. Since the HAR1 is extremely similar in non-human vertebrates, we considered the chimpanzee sequence as a proxy of the ancestral sequence.

We use the CS^2^BP^2^-Plot to highlight the major similarities and differences among the predicted secondary structures for the ancestral, an archaic human (Denisovan) and the modern human HAR1. For convenience and comparison purposes, the planar graph representation of each secondary structure is also included.

**Fig. 2:**
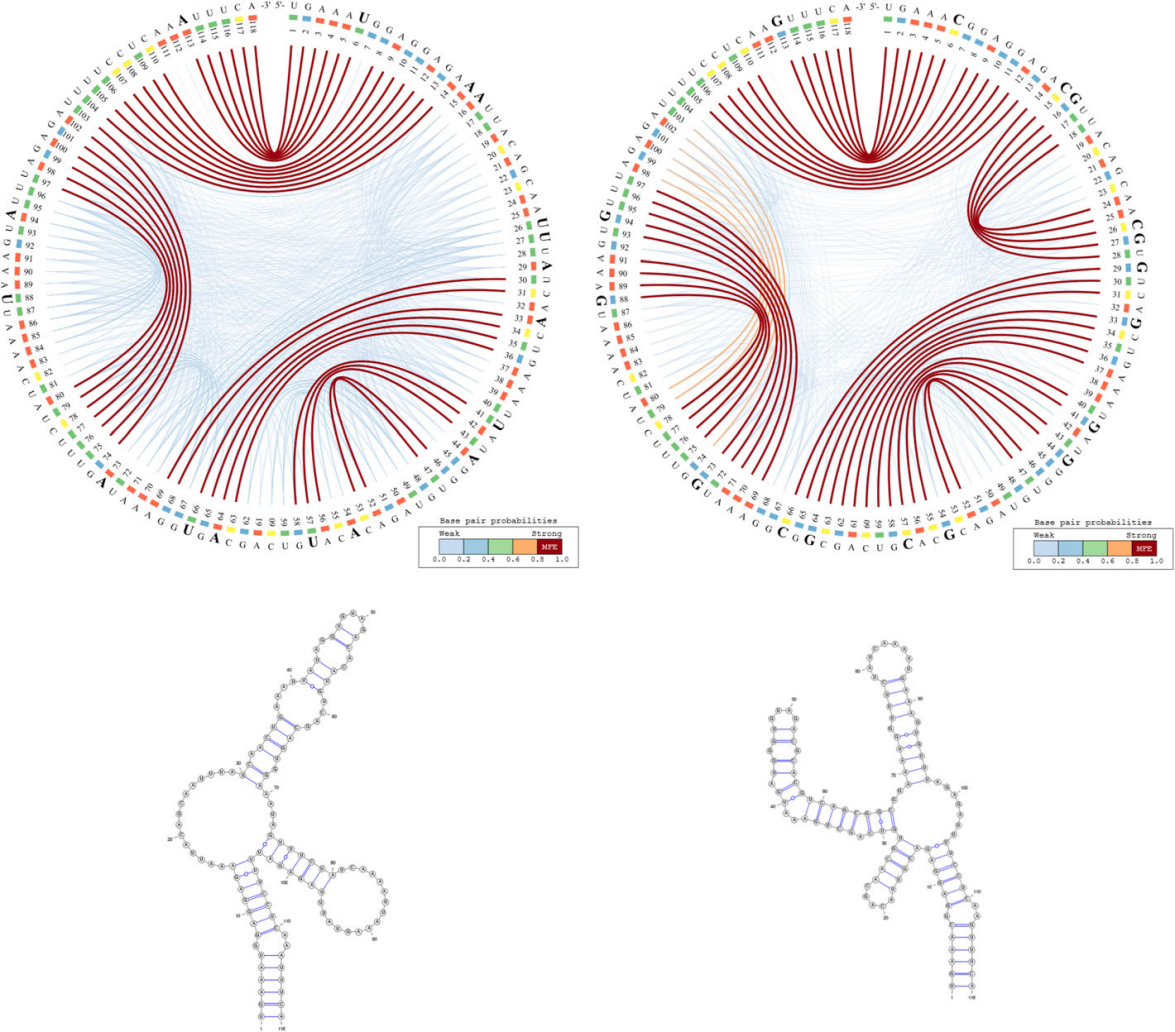
CS^2^BP^2^-Plot (top) and planar graphs (bottom) for ancestral (left) and Denisovan (right) HAR1 secondary structures. The CS^2^BP^2^-Plots include sequence differences highlighted in bold. The sequence differences are highlighted after we infer them from a ClustalW pairwise local alignment

**Fig. 3:**
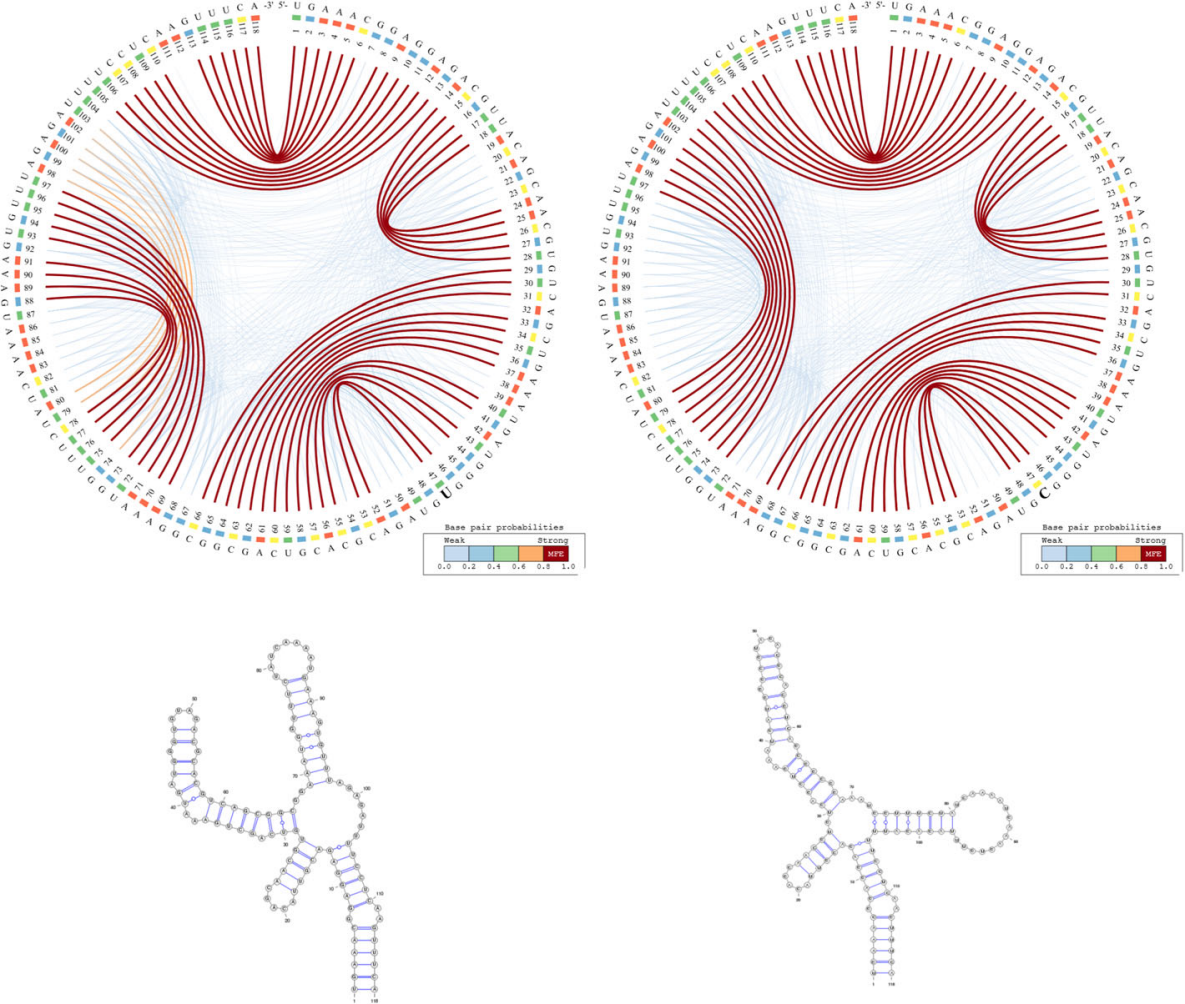
- CS^2^BP^2^-Plot (top) and planar graphs (bottom) for Denisovan (left) and Human (right) HAR1 secondary structures. The CS^2^BP^2^-Plots include sequence differences highlighted in bold

The information represented in Fig. 2 can be interpreted based on the predominance and the location of various visual cues related to colour and position. For example, the CS^2^BP^2^-Plot of the ancestral HAR1 structure (Fig. 2 – left side images) displays two internal sequences with no base pairings between positions 15 and 29 and between positions 81 and 96. By comparison, the corresponding internal sequence in the Denisovan (Fig. 2 – right side images and Fig. 3 – left side images) HAR1 structure contains two solid 4 base-pair stems formed due to 2 base pair mutations at positions 88 and 94 replacing the A and U bases in ancestral with the more stable Gs in Denisovan.

A total of 17 single and consecutive base pair mutations (positions 6, 15–16, 26–27, 29, 33, 41, 44, 54, 57, 64, 66, 73, 88, 94 and 113) can be identified between the ancestral and the Denisovan HAR1 structures, while only one mutation (U replaced by C) at position 47 occurred between the Denisovan and the modern human structures. The large number of mutations that distinguish the Denisovan from the ancestral sequences was apparently caused not only by the emerging of 2 new 4-base pair stems in Denisovan, but also caused by the breakdown of multiple existing stems in the ancestral, as well as the creation of new stems in Denisovan. The orange arcs in the Denisovan HAR1 CS^2^BP^2^-Plot suggest the existence of a powerful energetic pressure to create a stem between positions 74–80 and 98–103. This stem appears in the human HAR1 structure due to changes in the stem structures between positions 69–78 and 88–97. Importantly, the single mutation that differentiates the sequences of modern and archaic humans caused a stabilization of a stem that reverted back to the ancestral state. This observation is biologically very relevant, since it also corroborates the hypothesis that the HAR1 structure adapted in the human lineage towards an increase in stability. This is a novel piece of information that could not be acquired with the visual comparison of the classical dot-plots.

In a previous work on the HAR1 [19], we constructed a model showing that the most likely last step in turning the ancestral state into the modern human state was exactly the substitution at position 47 that distinguishes the modern human sequence from that of the Denisovan, which provides an independent support for the conclusions we drew from our visualizations.

We can also notice in the CS^2^BP^2^-Plot that the human secondary structure is by far the most stable with a total MFE_human_ = − 33.5 *Kcal/mol* out of all three HAR1 structures (MFE_ancestral_ = − 20.0 *kcal/mol*; MFE_Denisovan_ = − 32.1 *Kcal/mol*). This is likely due to the emergence of stronger base pairings in the modern human structure (low number of blue arcs), which could have occurred in the human lineage as a result of adaptive pressures.

**Fig. 4:**
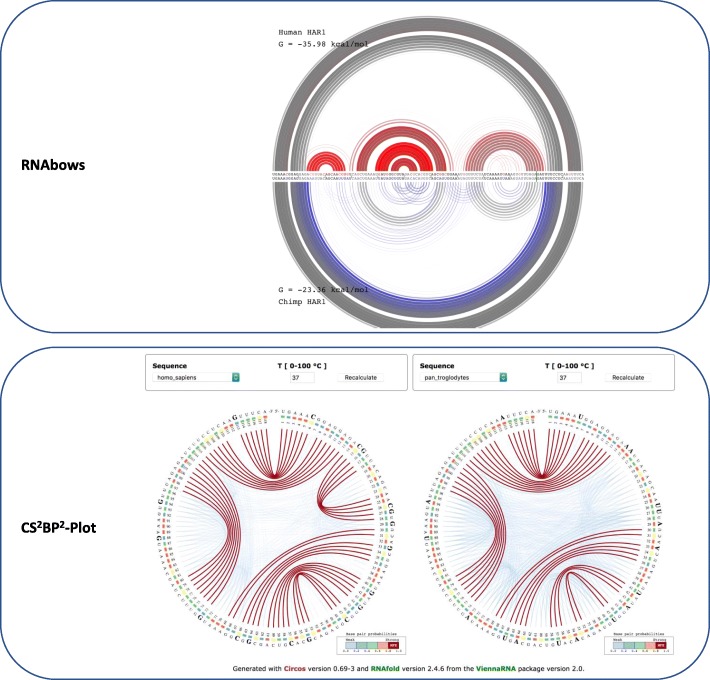
The RNAbows (top) and CS^2^BP^2^-Plots (bottom) representations for the human and ancestral HAR1 secondary structures. The RNAbows arc diagram was obtained using the diffRNABow functionality available at http://rna.williams.edu/rnabows/ using the Vienna-2.0.2 folding method. The CS^2^BP^2^-Plots were obtained using the default parameter settings available at https://nrcmonsrv01.nrc.ca/cs2bp2plot/

We also use this use case to show how the CS^2^BP^2^-Plot representation of human versus ancestral HAR1 ncRNAs compares with the RNAbows representations (Fig. 4). The stem structure differences between the two secondary structures of human and ancestral are explained in detail above and are much easier to identify when using the CS^2^BP^2^-Plot versus the RNAbows representation.

### Case study 2: visualization of energetic transitions between RNA secondary structures

The RNA folding process leading to a native fold with a minimum free energy is typically perceived as a succession of transitions between intermediate states or conformations that represent local minima in the free energy landscape. While the free energy barrier between two consecutive states of an RNA structure is typically small allowing for smooth and fast transitions, large energy barriers are sometimes encountered. These barriers act as folding traps and slow down the folding process, leading to bi-stable RNA structure. The first bi-stable RNA structure had around 150 bp in length and represented a ribozyme, which was reported in 2000 [20]. Since then, other studies proposed shorter RNA sequences (20–40 bp) with computationally proven bi-stable secondary structures [21, 22]. Nevertheless their experimental validation, based on UV-melting and gel assays, was not possible at the time due to their significantly fast timescale folding process. In 2003, Hobartner and Micura [23] successfully used ^1^H NMR spectroscopy to study a series of bi-stable RNAs of 18–20 bp in length, each RNA comprising of two competing stem-loop motifs.

The CS^2^BP^2^-Plot can be used to visualize the energetic transitions between two stable secondary structures of the RNA sequence 4 proposed by Hobartner and Micura [23].

**Fig. 5:**
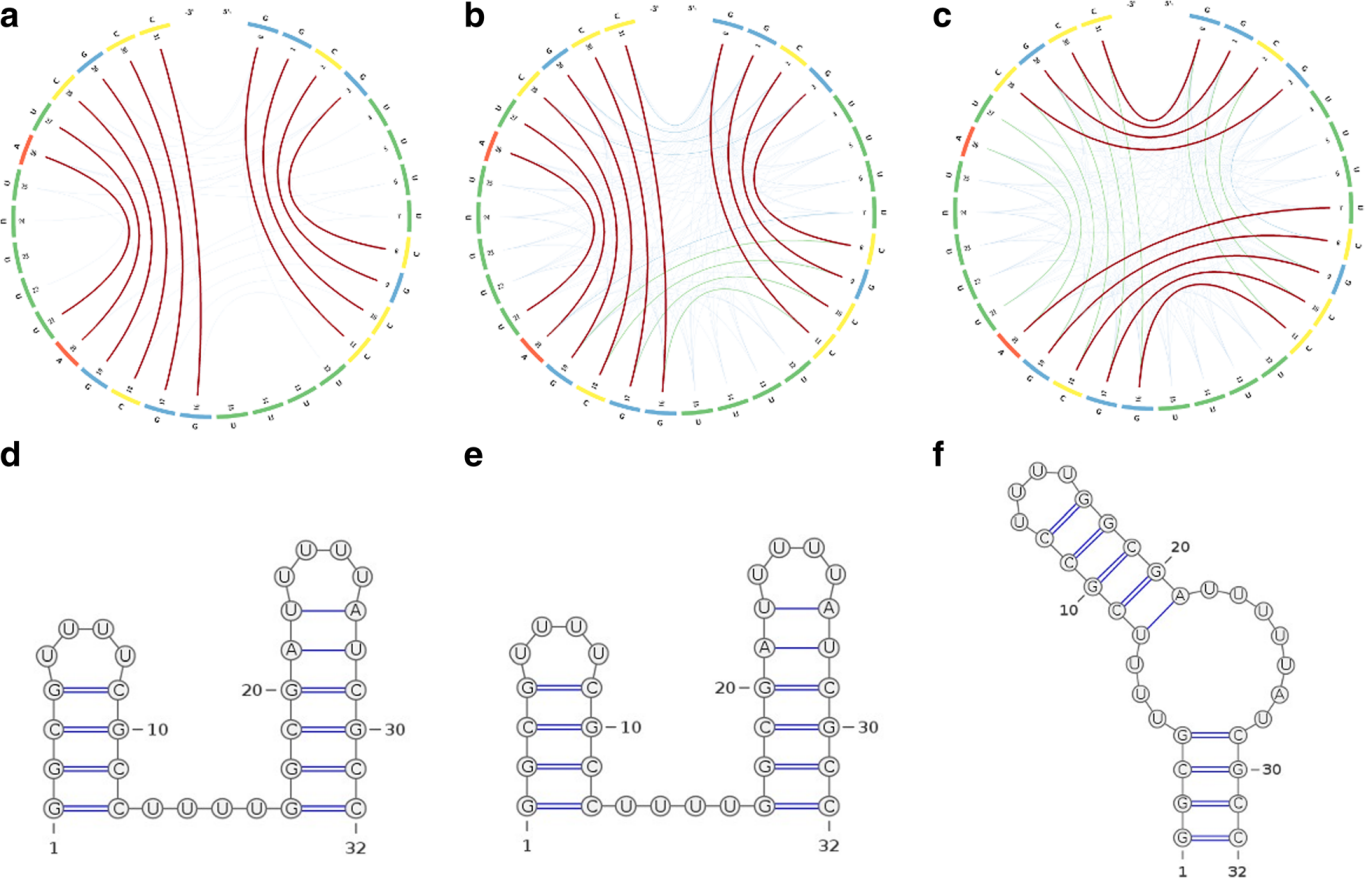
The CS^2^BP^2^-Plots (top) and planar graphs (bottom) for secondary structures of a small bi-stable RNA. Sub-figures (**a**) and (**d**) describe the secondary structure conformations at 25 °C. Sub-figures (**b**) and (**e**) depict the secondary structure conformations at 70 °C and sub-figures (**c**) and (**f**) depict the secondary structure conformations at 75 °C

Images (a) and (d) in Fig. 5 depict the secondary structure formed at 25 °C with an MFE = − 17.85 *Kcal/mol*. The two stems formed at positions 0–3/8–11 and 16–21/26–31 are in agreement with the reported 85–15 equilibrium by imino proton NMR spectroscopy. We can also notice that virtually no competing base pairs (no green or orange arcs) exist at this temperature. When the temperature is increased to 70 °C (images (b) and (e) in Fig. 5), two competing stems depicted by green arcs appear at positions 0–3/28–31 and 7–11/16–20 and the MFE of the secondary structure decreases to − 4.06 *Kcal/mol*. With further increase in temperature, the new stems will break the existing ones, thus forming a new secondary structure for the RNA sequence 4. Images (c) and (f) in Fig. 5 depict the second stable conformation of the RNA structure formed at 75 °C with MFE = − 2.63 *Kcal/mol*, which is supported by the UV-melting analysis reported in Hobartner and Micura [23].

### Case study 3: gRNAs for CRISPR/Cas systems

Guide RNAs (gRNAs) are typically used in CRISPR/Cas systems to direct sequence-specific DNA cleavage at desired locations along the target sequence. Nevertheless, gRNAs have a wide spectrum of cleavage effectivity. While there is a large amount of active research in the field, the mechanisms and factors governing their activities are still poorly understood.

Thyme et al., 2016 [24] suggest that there are two potential mechanisms that decrease gRNA performance: (i) weak gRNA sequence content that do not form active Cas9-gRNA complexes, and (ii) gRNAs with in vivo refractory target sites.

Here, we use CS^2^BP^2^-Plot to: (i) provide a visual interpretation that supports the hypothesis that gRNA secondary structure plays a significant role in the modulation of Cas9 cleavage efficiency, and (ii) suggest a third potential mechanism that might contribute to better gRNA performance, which is the need to design gRNAs with minimal self-folding structures.

**Fig. 6:**
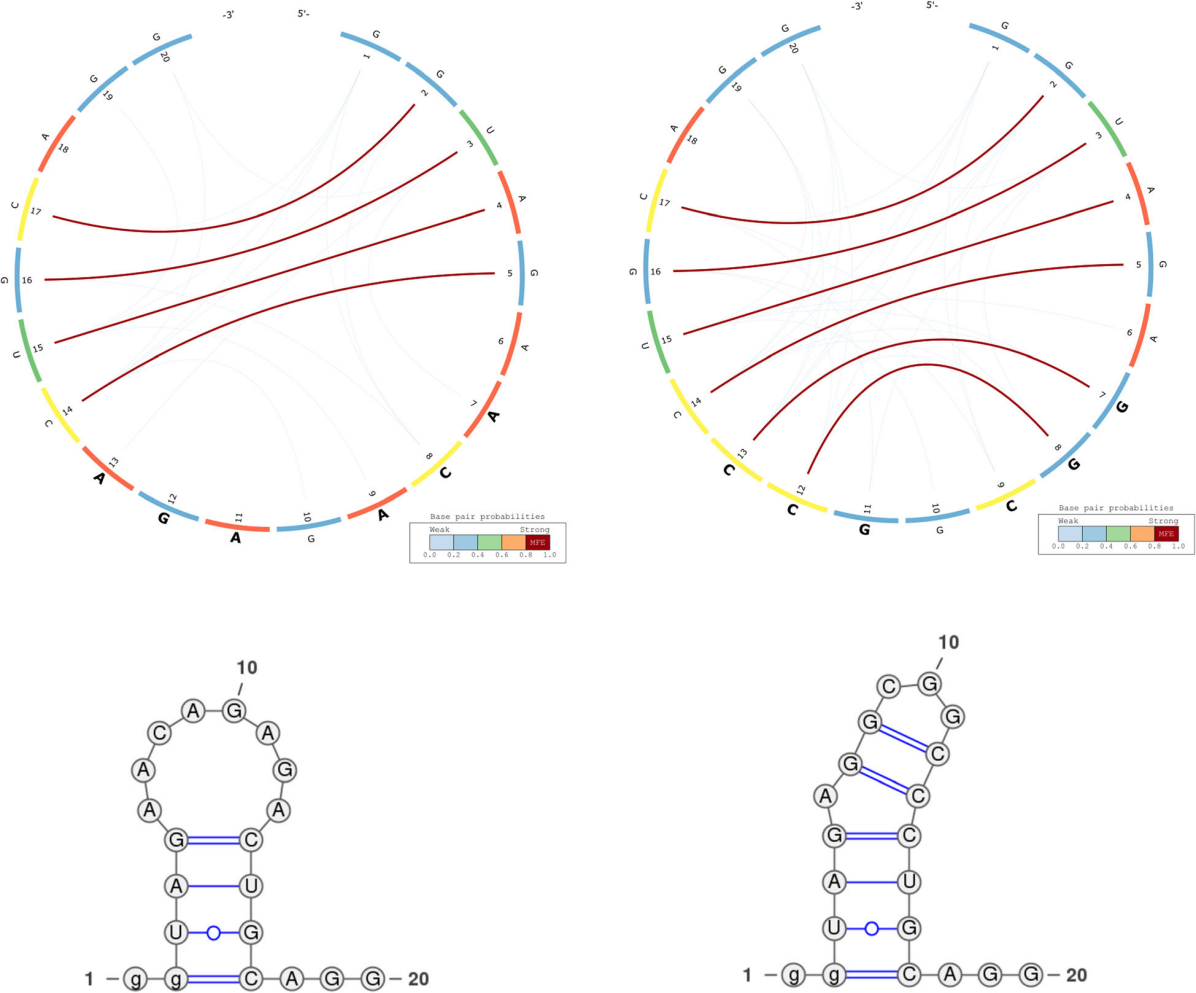
Active (left) and inactive (right) gRNAs from Thyme et al., 2016. The secondary structures of the gRNAs include a pair of non-canonical base pairs (G-U) at positions 3–16

Figure 6 depicts the predicted secondary structures of an active (left) and an inactive (right) gRNA sequence from Thyme et al. (2016). The inactive gRNA has a significantly more stable secondary structure (MFE = − 4.40 *kcal/mol*) compared to the active gRNA (MFE = − 2.6 *kcal/mol*). This suggests that strong hairpin formation hampers the ability of gRNAs to interact with the desired target, an effect that can be observed on all gRNA sequences and their mutated variants from Thyme et al. (2016).

### Case study 4: RiboSNitches

A RiboSNitch is a regulatory RNA in which a specific Single Nucleotide Polymorphism (SNP) has a structural consequence that results in a local or global conformational change in the secondary structure, which could lead to a disease phenotype.

Unlike a riboswitch, a RiboSNitch results in a permanent change in regulation and can thus lead to disease phenotypes. RiboSNitches represent a novel diagnose and therapeutic target, since small molecules can repartition the RNA structural ensemble [25].

**Fig. 7:**
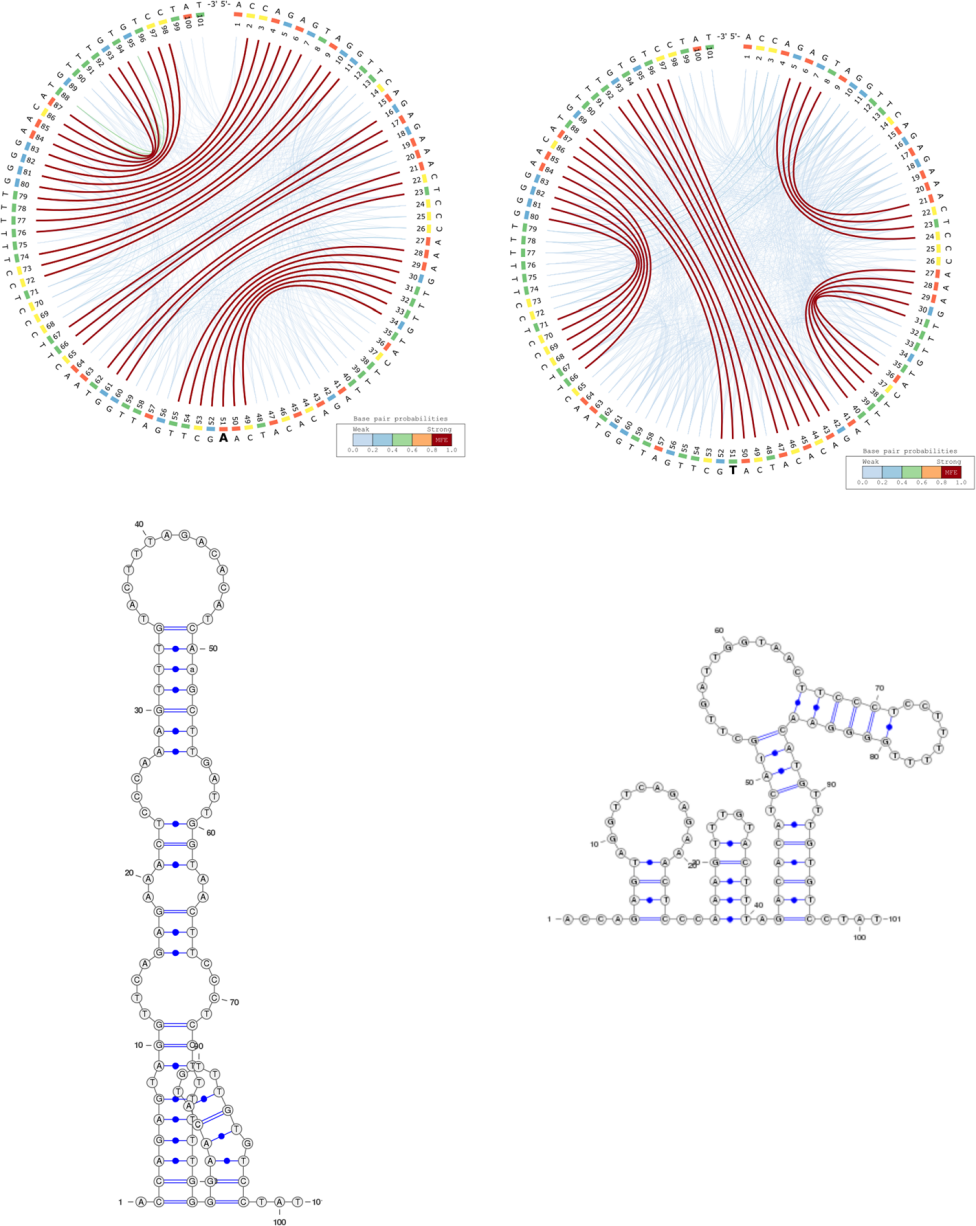
The CS^2^BP^2^-Plots and planar graph diagrams of the sequence flanking an SNV in the 3’UTR of SUB1. The activated RNA polymerase II transcriptional co-activator p15 or SUB1 homolog was previously identified as a riboSNitch by Corley et al. [24]

Here we use CS^2^BP^2^-Plots to computationally validate and visualize one of the riboSNitches presented by Corley et al. [26], which represents a sequence flanking an SNV in the 3′ untranslated region of the activated RNA polymerase II transcriptional co-activator p15 (SUB1). A single base mutation at position 51 has a major effect not only on the MFE structure, but also on the entire structural landscape of the RNA secondary structure as depicted in Fig. 7, which can be more clearly visualized with our new representation. In a similar manner, the CS^2^BP^2^-Plot could be used to investigate other putative RiboSNitches, providing valuable and intuitive visual clues about the impact of SNPs on the entire landscape of the structure.

### Case study 5: RNA thermometers

The RNA world is very diverse and their role in living organisms spans a large spectrum. Some RNAs are able to control gene expression in a temperature-dependent manner and are called “RNA thermometers”, such as those identified in the structurome of *Yersinia pseudotuberculosis* [27]. Righetti et al. (2016) identified two candidate virulence factors for this pathogen: *ailA* (attachment invasion locus protein) and *cnfY* (cytotoxic necrotizing factor). They reported the genome-scale landscape of RNA structures of the human pathogen *Y. pseudotuberculosis* at three physiologically relevant temperatures reflecting environmental (25 °C), host body (37 °C), and heat shock (42 °C) conditions at single-nucleotide resolution.

**Fig. 8:**
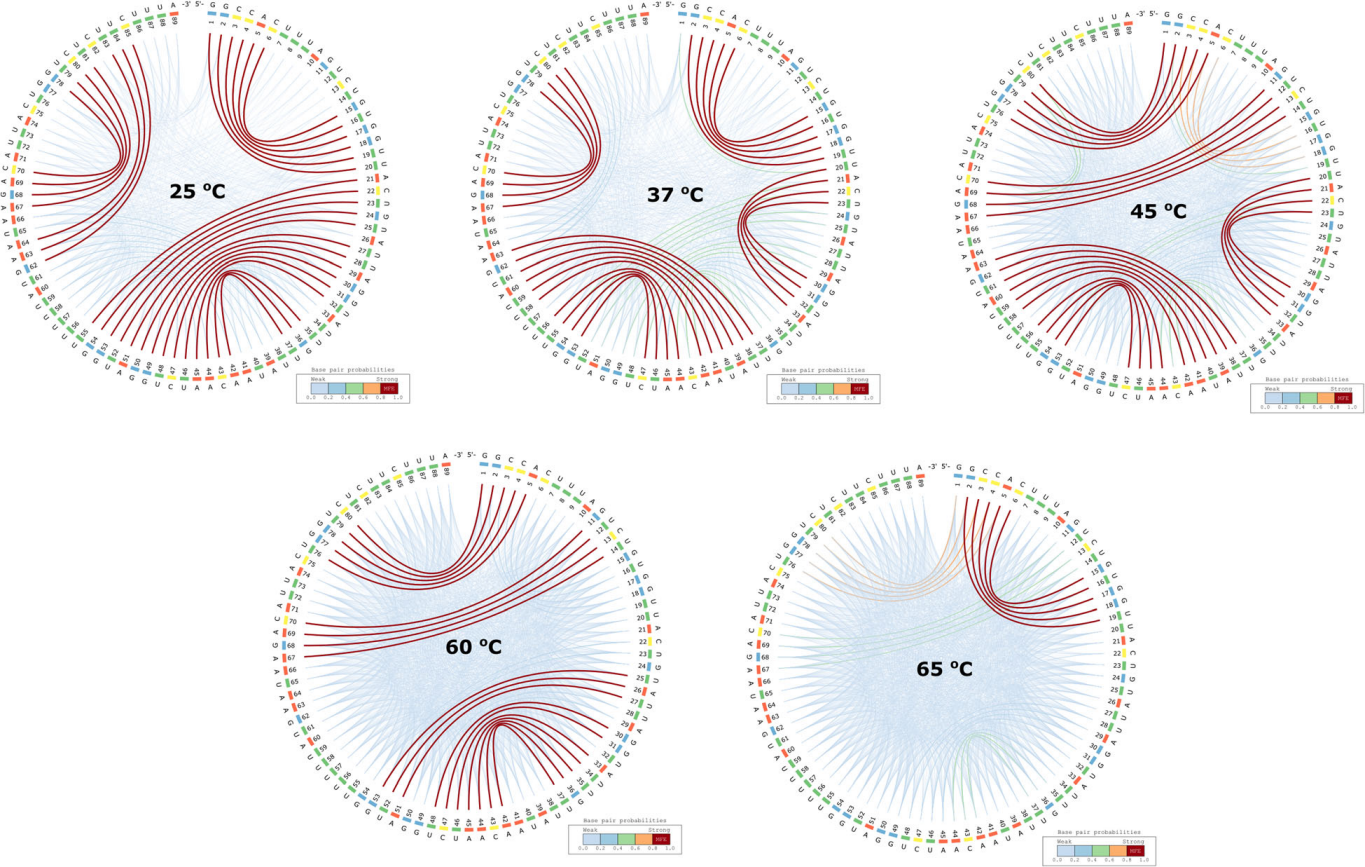
CS^2^BP^2^-Plots for *ailA* putative virulent factor in *Yersinia pseudotuberculosis* at five different temperatures. The *ailA* putative virulent factor from *Y. pseudotuberculosis* shows significantly different layouts of their secondary structures at 5 different temperatures: 25 °C, 37 °C, 45 °C, 60 °C and 65 °C

The CS^2^BP^2^-Plots and planar graph secondary structure representations from Fig. 8 are in agreement with the findings of Righetti et al. (2016), suggesting that the expression of virulence-relevant functions in *Y. pseudotuberculosis* and reprogramming of its metabolism in response to temperature is associated with a restructuring of some of its RNAs. We also performed *in-silico* experiments for higher temperatures (60 °C and 65 °C) and we observed a changing structural landscape for *ailA* by losing existing stems while forming new ones, which could qualify this molecule as a 5-phase riboswitch. Although experimental evidence is needed to further confirm these observations, our plots allow for a straightforward comparison of the landscape change when the temperature varies.

## Conclusions

Our results demonstrate that a single image consisting of circular diagrams combined with color and weight-coded arcs (the CS^2^BP^2^-Plot) can efficiently represent both the minimum free energy secondary structure and the base pair probabilities for an RNA structure. The CS^2^BP^2^-Plot significantly simplifies the interpretation, analyses and comparisons of RNA secondary structures, thereby making evolutionary and energetic transition results more easily understandable.

## Methods

The CS^2^BP^2^-Plot currently use Circos [28] as a graphical library and Go Language (https://golang.org/) scripts to prepare the input files for Circos. Nevertheless, other more versatile approaches could be considered in the future, such as the JavaScript D3 library, for enhanced interactivity and user experience. Our current input consists of up to 10 RNA sequences in FASTA format. In addition the RNA sequences were computationally folded with the aid of RNAfold from the ViennaRNA Package [6] version 2.0 at different temperatures. Nevertheless other RNA folding algorithms can also be used, such as Mfold [29], RNAsoft [30] and RNAstructure [31]. Default RNAfold parameters (*−-partfunc = 1, −-temp = 37, −-dangles = 2*) were used, with the exception of the bi-stable RNA secondary structures calculated at non-standard temperatures (25^o^ C, 70^o^ C and 75^o^ C).

Linear arc diagrams and planar graphs were generated with VARNA [3] version 3–93 using corresponding parameter settings (*−algorithm line, −algorithm circular*), while dot-plots were calculated with RNAfold version 2.4.6.

### The circular secondary structure base pair probabilities plot (CS^2^BP^2^-plot)

**Fig. 9:**
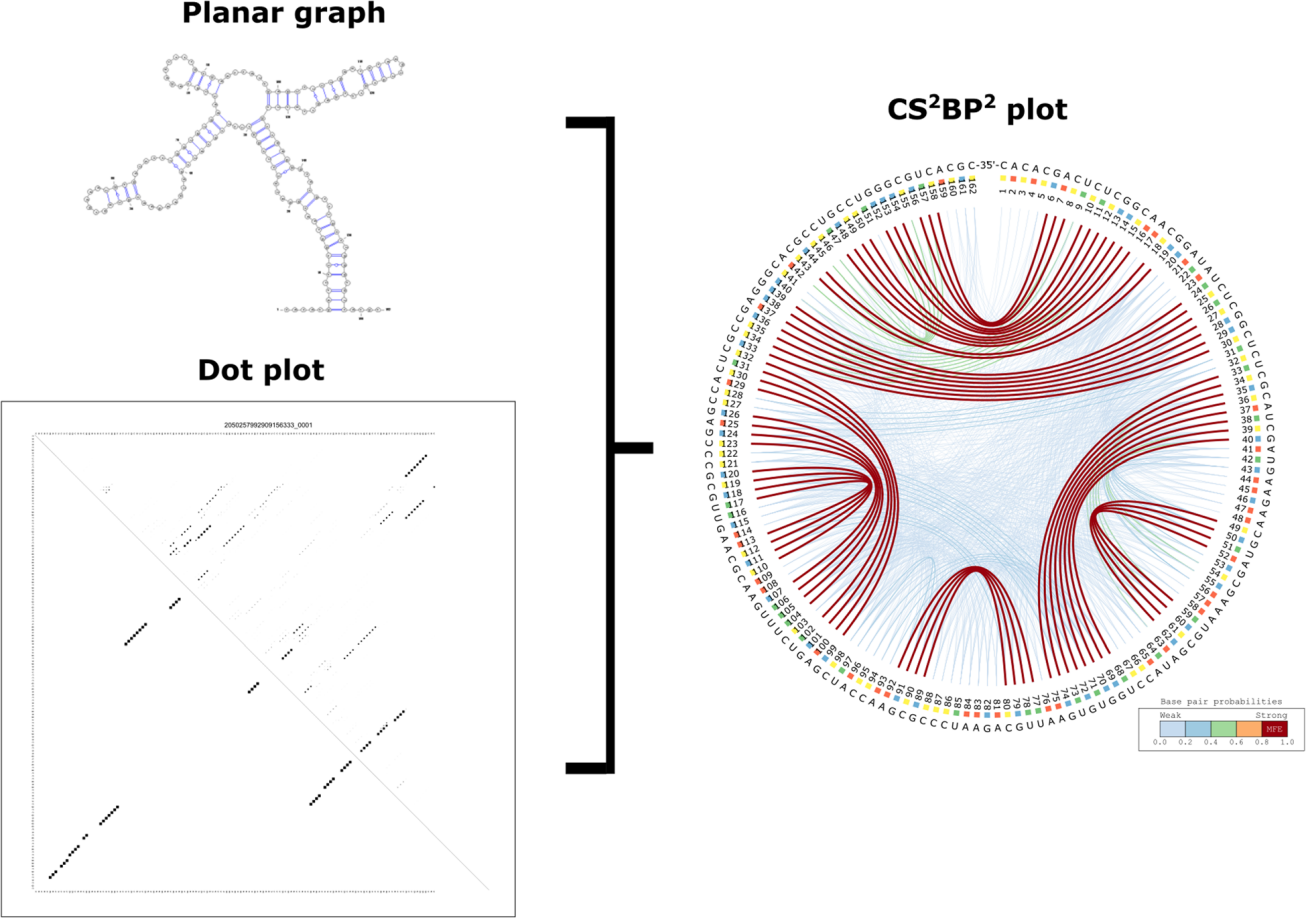
The planar graph, dot plot and CS^2^BP^2^-Plot of the nucleotide sequence of wheat-embryo cytosol 5.8S ribosomal RNA. The CS^2^BP^2^-Plot includes information typically included in two separate graphical representations (a planar graph for the minimum free energy base pairing configuration and a dot-plot for base pair probabilities)

The CS^2^BP^2^-Plot challenges the classical ways of representing RNA secondary structures and combines base pairings with dot-plot values in a single graphical representation (Fig. 9) capable of assisting biologists in quickly spotting similarities and differences among a large number of secondary structures and their corresponding RNA sequences.

The use of a circular diagram instead of a linear one is justified by the equivalent of π times savings in horizontal spread, where π (3.14 …) equals the circle length (i.e. the length of the RNA sequence) divided by the circle diameter (i.e. the width of the graphical representation).

The outer layer (the RNA sequence) consists of 4 types of equally spaced blocks colored and labeled corresponding to each base (A, C, G and U).

The inner layer consists of a set of colored arcs that connect base pairs on the RNA sequence showing hydrogen bonds depicted by short segments in a typical linear graph RNA secondary structure representation. The arc colours, currently using a 5-color palette assigned values within 0.2 unit intervals spanning the interval [0,1], are assigned based on the corresponding base pair probabilities, ranging from blue (less stable) to dark orange (more stable) with green representing base pairs with medium energetic stability. The thickness of each arc is also proportional with the uncertainty probabilities using 5 incremental sizes ranging from 1 (less stable) to 9 (more stable). The dark red arcs represent the *MFE* base pairing corresponding to the most stable interactions. Each plot includes a legend summarizing the probabilistic range significance for each color.

Originally implemented in Perl and currently converted to the Go programming language and using Circos version 0.69–3 [28] as graphical library, the CS^2^BP^2^-Plot can be applied to represent and compare secondary structures of various types of RNAs, such as small (< 200 bp) non-coding (ncRNAs) and bi-stable small RNAs.

### The CS^2^BP^2^-plot web server

**Fig. 10:**
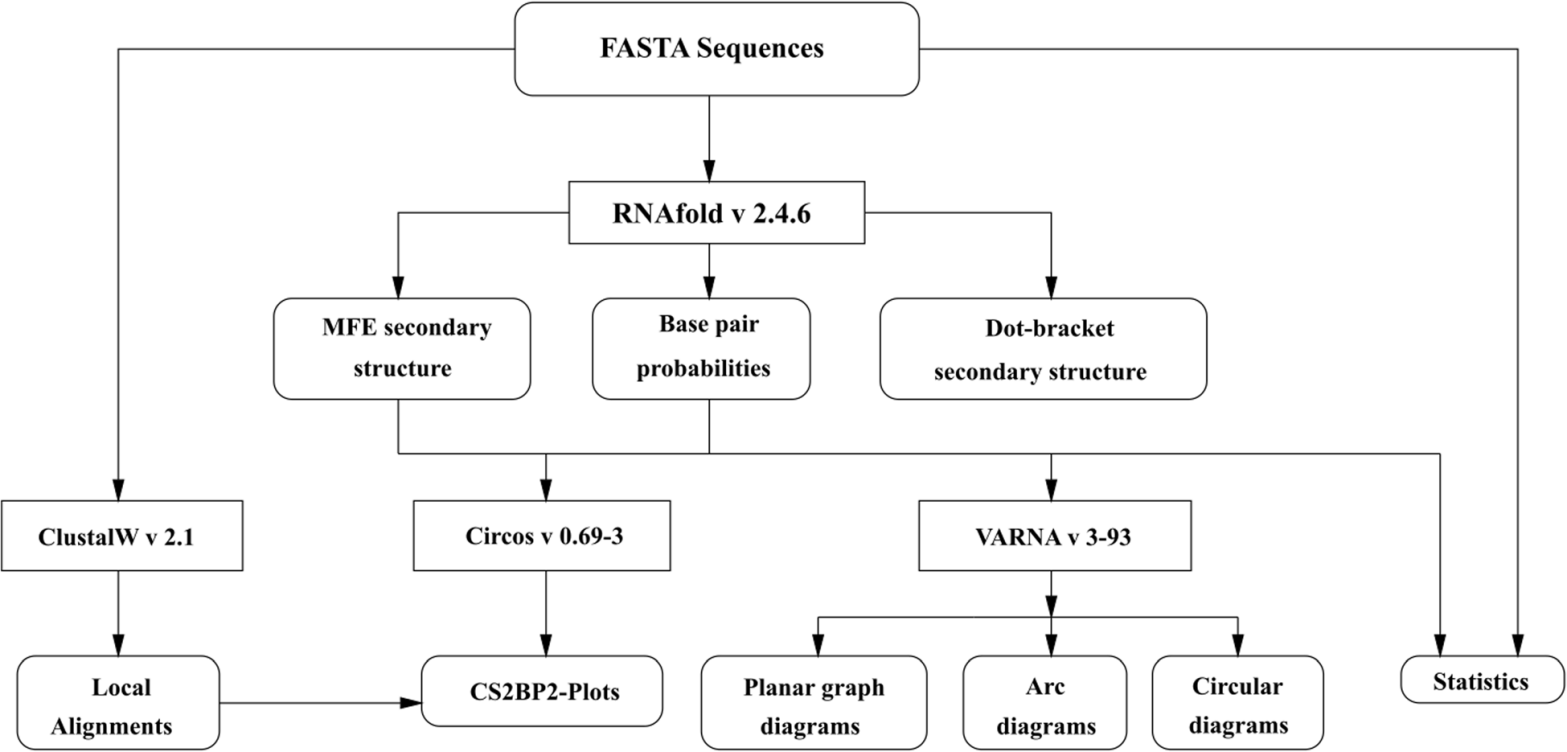
CS^2^BP^2^-Plot web server functionality flowchart. The web-server pipeline that implements and automatically generates the CS^2^BP^2^-Plots is depicted here. All data files (input and output) are depicted as round boxes, while the software used to process the files are described with rectangles

We provide a web server implementation that allows pairwise comparison of two predicted RNA secondary structures. As depicted in Fig. 10, the web server accepts as input up to 10 RNA sequences, each no longer than 200 bases and uses RNAfold version 2.4.6 from the ViennaRNA package version 2.0 [6] to predict their secondary structures and corresponding base pair probabilities. The user can adjust the temperature and 5 other parameters such as the linearity of the RNA molecule (linear or circular), G-quadruplex formation incorporation, forbidden lonely pairs, and forbidden GU pairs along the whole sequence or at the ends of helices.

Once the secondary structures and base pair probabilities are calculated for all sequences, two sequences are selected for visualization and their corresponding CS^2^BP^2^-Plots are generated using Circos version 0.69–3 [28] and a customized template design that allows for highlighting of base pair differences between two sequences based on a local alignment performed using ClustalW version 2.1. This provides a significant advantage over other secondary structure comparative tools such as diffRNABow [4], which work only for two sequences of the same length. In addition to this, we use VARNA version 3–93 to generate the corresponding traditional planar graph, arc and circular diagram plots for the convenience of the users that are more accustomed with this type of visualization.

The webserver also provides information regarding the sequence length and base content (single bases, AT/AU and GC), the MFE of the predicted structure, the number of MFE base pairs, the number of similar and different base pairs between the two sequences, and the dot-bracket representation of the MFE secondary structure. The information is structured so that each section can be expanded or contracted allowing the user to focus on the relevant sections. In addition, all the information generated by the webserver is downloadable as a ZIP archive.
